# Discussion

**Published:** 1904-02

**Authors:** Alphonso Irwin


THE AMERICAN

### Journal of Dental Science.

### DISCUSSION.

DR. ALPHONSO IRWIN.

I have attempted to make an analysis of Dr. Inglis's paper, and it seems to me he has called to your attention two methods of treatment; the first is immediate root filling in contradistinction to the second, or tentative method. It has been my practice to pursue the tentative method, and yet from what I have heard this evening I feel I must try the method presented by Dr. Inglis.

One of the main points brought out is the desirability of immediate root filling. This is largely to be considered in refereenc to the time limit of the patient and the limit of the operator. Another consideration which has been emphasized by Dr. Inglis is to devitalize the pulp either by the use of arsenic, coacaine or the use of electricity, and I can agree with him in the desirability of immediate root filling, provided such filling is removable.

Another point is that of instrumentation. We all know what an important feature in the treatment of nerve canals that is. The touch is one thing above all others which we have to cultivate, and the positive knowledge that we have removed from the root canal every possible particle of matter is a prerequisite to success in the treatment of root canals.

Another portion of his paper is properly placed under the head of sterilization, and he has suggested as his favorite germicide, eucalyptus, the essential oils and formaldehyde. He has also met with success in the use of deliquessed oxide of zinc, and also dilute chloride of zinc. In his statements in regard to this germicide or disinfectant I fully agree. There has also been brought to your attention the liability of infection. There are several sources of infection; there is the local source, the external source and another source to which I will refer as the infernal source. I have never met with the latter classification in any text-books nor heard any dentists speak particularly upon that point, but it is one on which we feel very keenly.

In conclusion, I think the whole secret of success in practicing immediate root filling, with a removable root filling, lies in the fact that the root canal is absolutely and perfectly cleanesd from the apex to the pulp chamber and that the canal is filled solidly with a permanent filling from the apex to the orifice of the cavity. Some men will say, "If you plug up the foramen the teeth will be all right; there will be no apical infection." They will drive that truth home, and that seems to be, in their minds, the only essential to every operation and the work is secondary. Another man will assert that the source of infection can be from the dental tubuli and that septic matter will enter the canal and cause trouble.

As I have already stated, the secret of success in root filling, and in immediate root filling in my judgment, is the absolute and perfect removal of all debris, the perfect sterilization of the canal and the perfect filling of the canal from the apex to the floor of the cavity.

Dr. John Curry: There is but little left to be said but many things to be done in the matter of successfully carrying to completion the directions which Dr. Inglis has given us tonight. I accept the essayist's definition of what he calls "immediate root filling;" I consider his methods conservative and subscribe to them. If I make a mental reservation, I do not think it will be any more than the Doctor himself has done, nor any more than most of you will do. The matter of immediate root filling, as the gentleman who preceded me has said, is a subject which concerns dentists vitally, and I think I may be permitted to add that it concerns the patient vitally. The patient who comes perhaps three times a week for five or six months, to have a root canal treated, certainly ought to have something to say on this subject. Unfortunately they are not here, but we are their proxies.

As Dr. Irwin said, one of the important features of success in this method is complete sterilization, and when we speak of that we mean as complete as we can obtain. I do not think any dentist is ever absolutely positive in his own mind that he has fully and completely sterilized a root, even when he hopes that he has. In the majority of cases, however, sterilization can be carried forward so successfully and thoroughly that we can safely stop. Suppose we should have some apical irritation, local applications to the gum or counter irritation will remove it. If we have an abscess and we have succeeded in getting our root dry, that is the time to fill the root. If no inflammatory symptoms supervene, it is a very quick and ordinarily a painless operation to perform the alveolar process and so relieve the distress. Of course you do not tell the patient exactly what you are doing, for that might terrorize hi ma little if he knew you were going to drill through the bone. I never go into details until after the operation is over.

The matter of time is, in my mind, one of the most important considerations concerning this method. Unfortunately the American dentist is not like the Spanish practitioner. We have no *"Mannana:"* we are working every day in the eternal "now," and are doing as much in one day for our patients and ourselves as we can. While the rubber dam is over the tooth and the patient in the chair, it does not take very much longer to make a thorough operation than it would to clean out the canal a little, put in some cotton and tell the patient to come again the next day. We all know that the receiving and dismissing of patients are great time consumers.

So, from the standpoint of surgical asepsis, and from almost any other standpoint from which one can view the subject, I would subscribe to the method which the essayist has so clearly and concisely set forth.

Dr. Joseph Head: There were some points the essayist made that seemed to be especially valuable, particularly that which had to do with the cocainization of the nerve by pressure. This method, and the resultant immediate filling of the root canal, is extremely happy inasmuch as, if the nerve is free from septic poison and asepsis is observed with the instruments, according to the laws of modern aseptic surgery, there ought to be absolutely no reason why such a tooth should give trouble any more than that trouble should arise in any of the ordinary abdominal operations where proper aseptic care has been used. It is one of the few operations where proper aseptic care has been used. It is one of the few operations in dentistry in which we feel that absolute asepsis can be obtained. But there is a special advantage in this method in destroying the pulp in molar teeth, by pressure with cocaine, that perhaps has not been brought out. If, for instance, we make entrance into the pulp of a molar and make pressure with cocaine, and presumably get out all the pulp that is there, we can on the following day have an absolutely certain guide as to whether we have taken out all of the pulp or not. As we know, the pulps in molars have two, three or more branches, according to the structure of the tooth, and it is occasionally extremely difficult to tell by working with a drill and by observation, whether all of these canals have been discovered, but if cocaine has been used and the pulp found and removed, on the following day the effect of the cocaine will have passed off. Then if pressure no longer gives pain, we may be sure that all of the pulp canals have been found, and the nerve filaments in these canals extirpated; but if on examination and pressure we find the same sharp thrill that would be given if the nerve filaments were there, we may be quite certain that some of the pulp is left, which can then be traced and eradicated. This seems to me to be an essentially valuable point and one which in my opinion gives extirpation by cocaine pressure, a certain precedent over the old arsenic method.

Dr. Inglis also spoke of the use of chloride of zinc. That appealed to me very strongly. In some cases of blind abscesses, where there is a cavity in the jaw at the tip of the root, it is next to impossible to create an antiseptic condition. If it happens to be in a tooth where the tip can be easily reached through the alveolar process, unquestionably the surgical procedure of drilling through the alveolar plate is the best way, as it can be opened and thoroughly cleansed and the filling inserted at once. But we all know that it is rather difficult to reach the tip of the lower twelfth year molar, or wisdom tooth, or even the lower sixth year molar, and while of course the surgical method is feasible with the upper teeth, where the external plate is thin, with the lower molars we have to resort to other means to destroy and get rid of the infested cavities which are the prime causes of blind abscesses. In my opinion the best means of destroying these is to inject through the apical foramen a small portion of delequescent chloride of zinc. It will create a very severe pain for the time being, but it will cause granulations to fill the cavity. It is true that when you do it you had better select a patient who is weaker than yourself or else get into a fireproof safe and stay there for the next twenty-four hours. But at the end of that time the patient will come and call you blessed because the tooth will have been saved which might otherwise have required extraction.

Dr. Ridgeway: I would like to ask the essayist if he has ever used oxyphosphate after the pulp has been taken out, working it into a canal.

Dr. Inglis: With regard to the time limit I would like to say that my idea was not altogether the saving of time. In many cases the treatment of putrescent root canals by means of the tentative method seems to result in a worse condition than it was in even at first. Not infrequently after treating the canal tentatively, I have found pus coming from the apical tissue. Perhaps in those cases I had not carried the original sterilization as far as I would have done had I intended immediate root filling. It may be that the upper or apical portion of the canal still contained some septic matter, and that when the antiseptic, such as the oil of cassia or the oil of cloves or any other antiseptic of that nature, or even some of the aqueous antiseptics, were placed in position, a thorough sterilization was not effected. The time limit of course is a valuable consideration and we can save hours of time in using such a material as forma-percha. If that material is an antiseptic, and if you can thoroughly sterilize the root canal and then place that material as a temporary antiseptic or as a permanent one, just as the case may turn out, you are no worse In such cases you have at the second filling to fill the canal with a permanent filling at once, for even if it is not successful, you only have to repeat it?

Another point is the safety of removable fillings and under very many circumstances I think that it can be said that on the whole the removable filling is perhaps better than the permanent one.

Sometimes teeth will be comfortable for an indefinite time under very bad treatment. I removed the other day a very handsome plastic filling which had been in position for some twenty years, the work having been done by a gentleman who was formerly one of the best operators in Philadelphia. The pulp had been almost removed but some had been left in one of the roots, while the other roots had been filled and no trouble was felt during all those twenty years, although there was a dead pulp in position all that time, and I know it must have been treated at that time for the cotton dressings were in position in the root.

I am perfectly in agreement with Dr. Irwin concerning the importance of the removal of the entire pulp. Why leave anything in the canal to become putrescent and give you trouble in the future? If you can get it out it is your duty to do so, and if you cannot, then you have done the best you can. I firmly believe if a man will do the best he can mechanically and then place a good antiseptic filling in the canal, he has done as any one can and has done thorough work whether he perfectly fills to the apex or not and in nine-tenths of the treatment of such roots a perfect filling to the apex is a lost art, or it never existed.

There may be some cases in which it is impossible to attempt an immediate root filling; there are some cases in which you cannot stop the flow of pus at the first sitting, and it would not be warranted to place anything in the nature of a permanent filling at that time. It is better to use methods that will drain the tissue or you will need to resort to the method of going through alveolar process and forcing the medicant through the canal.

In regard to Dr. Head's remarks upon the cocaine pressure anesthesia, it is not my desire to have it considered in that way. That was a little apart from the subject of the paper. But the idea that Dr. Head brought out is a very good one, that by waiting until the second day one could obtain a knowledge of the presence of the vital pulp in the canal. Certainly that should be ascertained if there be any doubt. It is possible, though, in making pressure the second time, one would necessarily have to place the usual material in the canal and then, by making pressure, if a large apical foramen happened to be present I do not think he could possibly tell if a portion of the vital pulp existed, because very tender apical tissues—a very sensitive or irritated tissue—would be just as likely to respond, as a portion of the pulp. In fact only recently I had to remove a formapercha filling owing to the fact that I had made pressure on the apical tissues when I placed the filling in the root. It is a very plastic material and squeezes into all the interstices and there is no reason why too much pressure should not force a portion of the material through into the apical space. You are all aware that pressure upon the apical tissues is apt to result in traumatic pericementitis and n this particular case I noticed the patient winced a little when I made the pressure, but I determined to take the risk, and the next day I had to remove the filling. I am satisfied in that case it was entirely due to the pressure; for as I say, I noticed response to the pressure by the patient at the time of the filling.

Regarding the point of leaving nerve filament in the canal it is of value to remember Dr. Bogue's case which he presented some years ago, I think to the New York Odontological Society, at any rate at one of the New York meetings. He spoke of a case in which cataphoresis had been used in the removing of the pulp for a right molar and nearly all of the pulp was removed from some of the canals and a large portion from the others. Immediately after the removal of the pulp the case developed neuralgic symptoms and I believe the lady underwent an operation which involved the antrum and there was a good deal of medical treatment indulged in with no success. Finally the dentist opened one root of that molar and discovered the pulp was still vital. So it is important to remember in cocaine pressure treatment that vital pulps might remain in the roots which may cause neuralgic conditions.

My conception of the use of chloride of zinc was slightly different from that mentioned by Dr. Head, although I believe that chloride of zinc, in a more mild solution than the deliquesced, could be forced into a blind abscess, but I would not urge the use of deliquesced chloride of zinc in those cases, that is, forcing any quantity of it into the apical space. My suggestion was that in some cases where the apical tissue constantly exhibited serum and there appeared to be a chronic inflammation present, with the tissues relaxed and a constant escape of the serum or blood in the canal, to use the cotton repeatedly, constantly forcing swabs of it in the root and taking them out again at once saturated with the serum, thus showing that the serum has rushed into the canal immediately upon the withdrawal of the cotton; the use of chloride of zinc in such cases is intended as an astringent. It usually acts like magic in those cases, but on some occasions has failed.

I think Dr. Irwin brought out the point that I preferred oil of eucalyptus and formaldehyde as a germicide. I did not intend to say that; the germicide I mentioned in preference was sodium-dioxide, which is a most marvelous agent. Pyrozone, 25 per cent, is also valuable.

Concerning the use of oyyphosphate I see no reason why it should be used as a permanent material when you have so good a material as oxychloride of zinc, which remains plastic for a greater length of time and which makes a permanent root filling which most operators seem to think the very best. In addition to that the oxychloride of zinc is much more easily manand in a root canal and you canp ack it by swabs of cotton far better than you can oxyphosphate.

